# Effect of Resin Type on the Tribological Properties of a Three-Dimensional Self-Lubricating Composite Surface

**DOI:** 10.3390/ma11040643

**Published:** 2018-04-22

**Authors:** Dongya Zhang, Xizhou Sun, Kai Dang, Fen Gao, Hui Zhang, Guangneng Dong

**Affiliations:** 1 Key Lab. of Manufacturing Equipment of the Shaanxi Province, Xi’an University of technology, Xi’an 710048, China; sxzh6838@163.com (X.S.); Xutjxdk@163.com (K.D.); gf2713@163.com (F.G.); 2 School of mechanical engineering, Xi’an Jiaotong University, Xi’an 710049, China; zhanghui7@xjtu.edu.cn (H.Z.); donghjxjtu@126.com (G.D.)

**Keywords:** composites surface, polymer, high temperature, tribological properties, transfer film

## Abstract

In this paper, three kinds of polymer, of epoxy resin (EP), phenolic resin (PF), and unsaturated polyester (UP), were used as fillers to prepare the laminated composite surface, and the tribological properties of a composite surface were studied under dry sliding condition. The results showed that: (i) the composites surface without MoS_2_ exhibited high friction coefficient and high wear rate at 25 °C, while the friction coefficients were reduced when the temperature increases to 100 °C; (ii) with the addition of MoS_2_, the friction coefficient of the epoxy resin composite containing MoS_2_ (E1) was below 0.22 under a temperature of 25–150 °C, and the friction coefficient was increased to 0.32 as temperature increased to 150 °C, while the average friction coefficient of the unsaturated polyester composite containing MoS_2_ (U1) was very low and below 0.20 under a temperature of 25–150 °C. Analysis of the wear scars indicated that, for the MoS_2_-containing composite, the transfer films of the E1 and U1 were smooth and continuous under low temperature, while the transfer film of U1 was comparatively complete than that of E1 under 150 °C. The composites with solid lubrication had excellent high-temperature self-lubricating properties, which was attributed to the synergistic effect of the laminated structure, and the thermal expansion of the polymer, and finally a transfer film was formed on the sliding path.

## 1. Introduction

Solid lubricating coating is an important area of tribology and has been developed from single to a multi-components to meet the high requirements of harsh working conditions. The composite polymer coating has excellent and comprehensive lubrication performance, and can be applied under high temperature, overload, current-carrying permeability, and vacuum conditions [1]. However, major failures of the solid polymer lubricating coatings are caused by poor wear resistance. 

Three-dimensional composite coating combined with the solid lubricant and porous structure can improve the wear properties, dues to the lubricant stored in the porous substrate, and thermal expanded to the surface during friction process to supply the lubricant and, thus, the formation of transfer film [2]. Dai [3] filled MoS_2_ and PTFE into porous copper to prepare the self-lubricating composite; the friction coefficient was 0.11, and the wear rate was 2.52 × 10^−5^ mm^3^/N m, which was reduced by 18% and 50% compared with the lubrication coating without the porous skeleton. However, the pore size of the matrix was random and hardly precisely controlled, and the surface texture technology is a technique that fabricates micro-dimple, micro-groove, and/or other regular morphologies on the surface [4].

The combination of surface texture and solid lubricating technology has been widely used to store lubricants. Rapoport [5,6] filled MoS_2_ and MoSe nanoparticles into micro-reservoirs produced by laser surface texturing, and the tribological properties of the composite surfaces under dry friction was studied. Hu [7] fabricated the surface texture on a titanium substrate and filled MoS_2_ in the dimples; the friction experiment showed that the textured surface filled with solid lubricant could significantly improve the lubrication performance. Mshkovith [8] processed texture and filled MoS_2_ particles in the surface, the lifetime of textured composite surface was increased more than two times compared with the traditional lubricating surface, and the friction coefficient also decreased from 0.30 to 0.10. Li [9] utilized textured composite coatings with dimples and silver. The friction coefficient of the silver coatings on the textured surface with appropriate dimple density was lower and more stable than that on un-textured surfaces. To improve the friction and wear properties of ceramic tool material, Xing [10] fabricated surface textures combined with solid lubricants of MoS_2_ and WS_2_. It showed that a combination of surface textures and solid lubricants could effectively improve the tribological properties of ceramic surface and protect the counter-face of the ball. However, the depth of the dimples on the surface fabricated by laser etching or ion etching is shallow, and it is difficult to store a large amount of solid lubricant in the dimples. It is obvious that increasing the depth or the area density can restore more solid lubricant, but the supporting strength of the sliding surface is significantly weakened as a deeper dimple or larger area density is applied, which reduces the wear resistance of the composite surface.

Multi-laminated biomaterials, such as bamboo, and bivalve shells are selected by natural evolution for their strong load-bearing, high impact resistance and low-density properties. Laminated composite is a type of material designed based on the bionics principles of the seashell structure; the strong layers were steel and/or ceramic sheets and the weak layers were filled with solid lubricant. By adjusting the type and content of the components to achieve specific functions. Kim [11] obtained carbon epoxy laminated composites with a grooved surface and the composite surface significantly improved the wear resistance because a lubricating film was formed on the contact region during dry friction. Su [12] introduced a bamboo structure to fabricate high-performance Al_2_O_3_/Mo laminated composites. Results showed that the laminated composite had high fracture toughness and exhibited good friction reduction at 800 °C. The excellent mechanical and tribological properties of the material were attributed to the presence of a hierarchical architecture, significant deformation of molybdenum boundaries, load redistribution, and the formation of a continuous molybdenum oxide lubricating and transferring films at high temperature.

Additionally, we fabricated steel-polymer laminated composites and studied the tribological performances. It was found that the polymer with a 40% filling rate had better tribological performance [13], and the lubrication mechanism was simulated by using a thermo-mechanical model [14]. Meanwhile the tribological performances of steel-polymer laminated composites under high temperature [15] and a magnetic environment [16] were both discussed. However, the friction and wear behaviors of steel-polymer laminated composites on the type and geometry of the polymer, the type of lubricant fillers to be used, and the test conditions, were still not well understood and certainly still needed further exploration. Hence, full exploration of the lubrication behaviors of steel-polymer laminated composites and an understanding of their tribological applications under dry and high temperature conditions were surely meaningful and significant.

In this paper, three kinds of polymer epoxy resin (EP), phenolic resin (PF), and unsaturated polyester (UP) were selected as the filling matrix to prepare the laminated composited surface, and the tribological properties under dry sliding condition were studied. Meanwhile, MoS_2_ particles are widely used as a solid lubricant, and the effect of the solid lubrication particles on the friction and wear of the laminated composite surfaces were also discussed under elevated temperatures.

## 2. Materials and Methods

### 2.1. Preparation of Laminated Composite Surfaces

Three kinds of resins were used for fabricating steel-polymer laminated composites. They were epoxy resin (EP, with type E-44) and curing agent of 650-type polyamide resin, phenolic resin (PF) and its curing agent of sulfuric acid ethyl ester, and unsaturated polyester (UP), and the corresponding curing agent of methyl ketone peroxide, which were pushed from Sinopharm chemical reagent Beijing co. LTD., Beijing, China. MoS_2_ particles (obtained from Sinopharm, Shanghai, China) with the average particle size of 0.5 μm were used as self-lubricating materials in the experiment.

The formulation of the polymer composite is shown in Table 1. First, the resin, MoS_2_ particles were stirred thoroughly in a mortar, followed by grinding until solid lubricant particles were uniformly dispersed, a curing agent was added and stirred again for 30 min until the components were homogeneous. Five polymer composites were compounded, as shown the Table 1.

**Figure 1:**
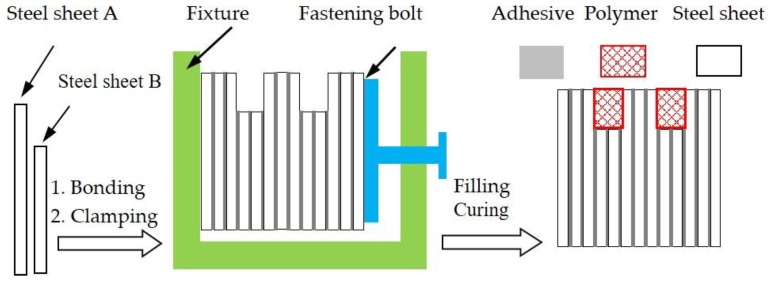
Schematic diagram of the fabrication of the polymer-steel laminated composite.

Polymer-steel laminated composite was fabricated by the bonding-laminating process. Steel sheets with a size of 40 × 30 × 0.35 mm (A) and 40 × 28 × 0.35 mm (B) acted as the substrates. The preparation process of the laminated composite is shown in Figure 1. (i) The steel sheets’ surfaces were polished with 400# metallographic sandpaper to increase surface roughness, and then were ultrasonically cleaned in acetone with petroleum ether and ethanol for 30 min and dried. (ii) Before laminating, polymer adhesive was sprayed on the two surfaces of the steel sheet. (iii) Setting five sheets as a lamination union, a surface with regular microgrooves was fabricated by laminating together different heights of silicon steel sheets (A:B = 3:2) [13], then the laminated composites were clamped and cured under 100 °C for 2 h. Then a polymer composite (as shown in Table 1) was filled into the groove surface, the polymer composite surface was obtained and cured at 120 °C for 2 h, and naturally cooled. Finally, the composite was polished with metallographic sandpaper to remove the polymer until exposure of the steel matrix, and the composite surface was polished to Ra = 0.1 μm.

### 2.2. Performance Test

The hardness of three resin matrices without adding MoS_2_ were measured by a micro Vickers hardness tester (TMVS-1, TIMES Group, Beijing, China). For the test conditions, the applied load was 0.49 N, and the test time was 15 s. All the adopted average values were tested at three different points.

**Figure 2:**
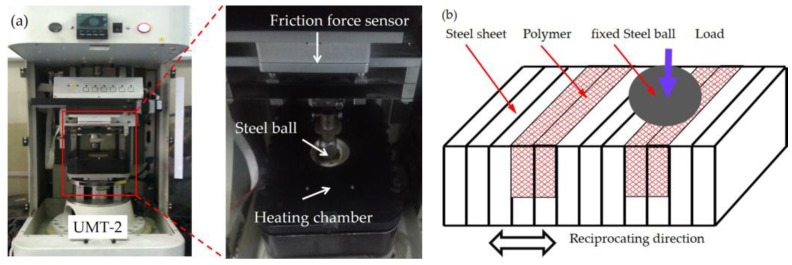
(**a**) UMT-2 multi-function tribo-meter and (**b**) the sliding direction.

Linearly reciprocating ball-on-disk friction and wear tests were performed on a UMT-2 tribo-meter (CETR Corporation Ltd., San Jose, CA, USA) at various temperatures of 25, 50, 100, and 150 °C. The upper samples were GCr15 steel balls (HRC: 60–63, Φ9.5 mm) and the counter part was the laminated composite. Before each test, the ball specimens were ultrasonically cleaned with acetone for 10 min and dried in hot air, and the polymer composite was cleaned with alcohol wipes. The test conditions are listed in Table 2, and the sliding sketch is shown Figure 2. For high-temperature tribological testing, a heating chamber was fixed on the UMT-2 tribometer, where the heating chamber was heated by an induction heating coil. Before testing, the sample was fixed to the bottom of the obturate heating chamber using bolts, the heating was stopped until the sample temperature reached the set temperature, then the tribological test was carried out at the maintained temperature.

After the friction test, the morphology of the worn surface was observed by scanning electron microscope (SEM, JEOL JSM-6460, Tokyo, Japan), and the chemical component of the worn surface was examined by INCA Energy+ EDS (Oxford-instruments, Oxford, UK).

Wear rate was used to evaluate the wear resistance of the composite surface. The wear scar section profile can be outputted by the profile-meter. From the profile curve the scar width can be measured. The average width of the wear scar was obtained by measuring each wear scar five times. The wear volume was integrated based on the section width. The wear rate *k* (mm^3^/N·m) of the specimen was calculated as follows:

(1) $$k=\frac{\lambda l\left(r^{2}\times \arcsin\frac{b_{1}}{2r}-\frac{b_{1}\sqrt{4r^{2}-b_{1}^{2}}}{4}\right)+(1-\lambda )l\left(r^{2}\times \arcsin\frac{b_{2}}{2r}-\frac{b_{2}\sqrt{4r^{2}-b_{2}^{2}}}{4}\right)}{P\times S}$$

where *r* is the radius of the steel ball (4.75 mm), *λ* is the filling ratio (0.4), *l* is the linear stroke (6 mm) and b_1_, b_2_ are the wear scar widths (mm) of the polymer and steel sheet, *S* is the sliding distance (m), and *P* is the applied load (N) used in the test.

## 3. Results

### 3.1. Characterization of Polymer Composites

**Figure 3:**
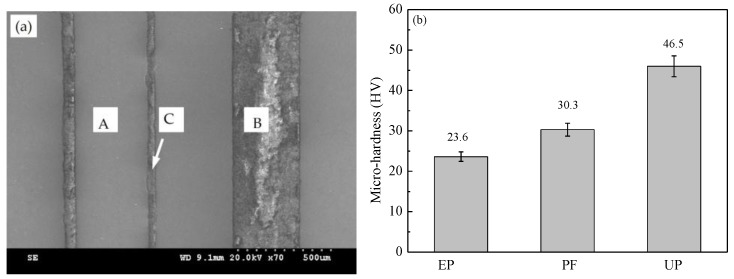
(**a**) Microstructures of laminated composite surfaces without adding MoS_2_ particles; and (**b**) the hardness of the polymer surfaces without adding MoS_2_ particles.

The microstructure of the laminated composites’ surfaces without adding MoS_2_ particles are shown in Figure 3a. Marker A is the zone of the strong steel sheet, marker B is the zone of the filled polymer composite, and marker C is the zone of the adhesive layer with a width of 20–30 μm. This shows that the layers are tightly bonded and the laminated composites have a continuous uniform appearance. Figure 3b shows the micro-hardness of the polymer without adding MoS_2_ particles. The lowest hardness of the pure epoxy coating is 23.6 HV, while the hardness of PF is 30.3 HV, which is 28.3% higher than that of EP. The highest hardness of UP is 46.5 HV, which is two times that of EP, which indicates that UP may have better wear resistance.

### 3.2. Tribological Properties of E0, P0, and E0 at Room Temperature 

The effect of the sliding speed on the tribological properties of the laminate composite surface sliding against the steel ball was investigated under sliding speeds of 4 Hz and 12 Hz and a load of 6 N at 25 °C.

**Figure 4:**
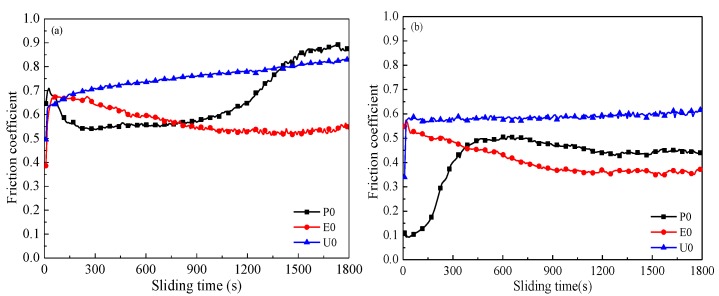
Friction coefficient under sliding speeds of: (**a**) 4 Hz and (**b**) 12 Hz and a load of 6 N at 25 °C.

The friction coefficient under a sliding speed of 4 Hz is shown in Figure 4a. This shows that the friction coefficient of P0 increases rapidly to 0.7 in the initial stage, and then declines and stabilizes at 0.53 after sliding for 300 s, then it smoothly increases after sliding for 1200 s and steadily increases. For E0, the initial friction coefficient is 0.4 and then increases to the highest value of 0.67, then it gradually decreases with the continuation of the sliding time, and the friction coefficient reduces to 0.55 after sliding for 1800 s. In contrast, the friction coefficient of U0 is 0.7 at the starting stage and then slowly increases with the prolonged sliding time, it reaches 0.8 at the end of the test. This indicates that the friction coefficient of U0 is higher than the other polymer composite surfaces.

Figure 4b shows the friction coefficient curves of three composite surfaces under a sliding speed of 12 Hz. Among them, the friction coefficient of U0 is 0.6 and remains stable during the whole test. The starting friction coefficient of E0 is 0.55, which then gradually reduces, finally achieving a stable value of 0.4 after sliding for 1200 s and then remains steady. Interestingly, the friction coefficient of P0 is 0.1 and then rises as sliding continues, stabilizing at 0.45 at 1350 s. It is obvious that the friction coefficients of three laminated composite surfaces are significantly lower than their corresponding friction coefficients under sliding at 4 Hz. It is generally recognized that the generation of frictional heat at higher sliding speed is more than that under the low sliding speed of 4 Hz. The polymer thermally expanded from the grooves under sufficient frictional heat, so it can quickly adhere to the steel ball and grind on the contact zones. Thus, a transfer film formed on the frictional surface [17]. This indicates that high sliding speed can generate more frictional heat in a short time, and that the polymer overflows quickly and forms a transfer film.

**Figure 5:**
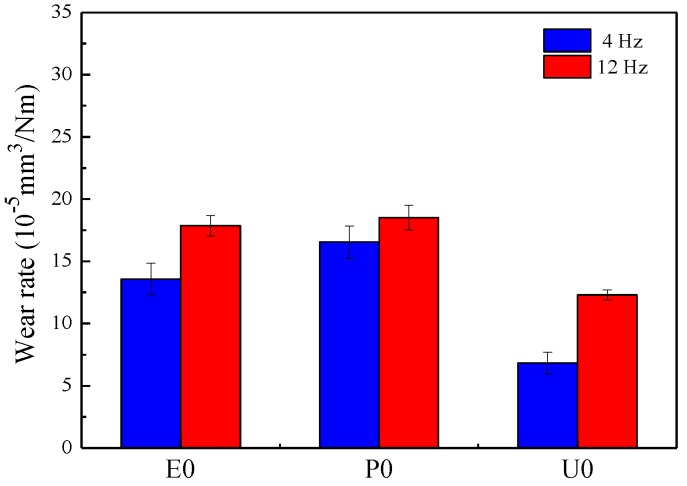
Wear rates of laminated composite surfaces under 4 Hz and 12 Hz at a load of 6 N and 25 °C.

Figure 5 shows the wear rates of the laminated composite surfaces sliding against the steel ball at 25 °C under sliding speeds of 4 Hz and 12 Hz. The wear rates of U0, E0, and P0 are 6.84 × 10^−5^ mm^3^/N·m, 13.58 × 10^−5^ mm^3^/N·m, 16.55 × 10^−5^ mm^3^/N∙m, respectively, at 4 Hz. Due to U0 with higher hardness, its wear resistance is better than that of E0 and P0. In addition, the wear rates of U0, E0, and P0 at 12 Hz are 1.80 times, 1.30 times, and 1.12 times higher than that at 4 Hz, respectively. This is mainly due to the high thermal expansion coefficient of EP and PF. A large amount of polymer filled in the groove is thermally expanded under the combined effect of high temperature and high sliding speed, which then forms a transfer film on the sliding surface. However, under higher temperature, the transfer film was rapidly consumed, resulting in a high wear rate under higher sliding speeds [14]. The hardness of UP is higher than EP and PF, and the polymer in U0 does not easily form a transfer film on the sliding surfaces, leading to a high friction coefficient. Plastic deformation easily occurs in the polymer in P0 and E0 and forms a transfer film; thus, their friction coefficients and wear rate are relatively low.

### 3.3. Tribological Properties at 100 °C

The tribological properties of the three laminated composite surfaces sliding against the steel ball under a load of 6 N and a sliding speed of 4 Hz at 100 °C were studied.

**Figure 6:**
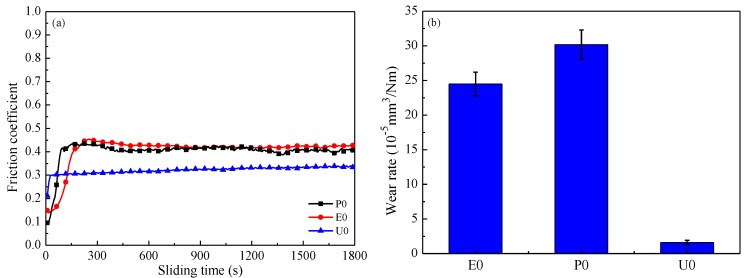
Friction coefficient (**a**) and wear rate (**b**) at 100 °C with a load of 6 N and a sliding speed of 4 Hz.

Figure 6a shows friction coefficient and wear rate of the laminated composite surfaces. The friction coefficient of U0 reaches a stable stage after running 30 s and maintains a low value of 0.24, while the friction coefficient of P0 and E0 are increased to over 0.42 after running 250 s, and then remains stable only with some small fluctuations. Compared with Figure 4a, the friction coefficients at 100 °C are much lower than that under 25 °C. This is mainly due to that the polymer has been heated to the surface before sliding test under 100 °C. When the polymer in the grooves is thermally expanded it forms a transfer film between two sliding surfaces, which is the major reason for the lower friction coefficient, and the polymer can effectively reduce the direct contact of friction pairs at a proper high temperature.

As shown in Figure 6b, wear rate of U0 is much lower than that of E0 and P0. In addition, the wear rate of U0, E0, and P0 are 0.23, 1.80, and 1.54 times of that at 25 °C. The thermal expansion coefficients of EP and PF are less than that of UP, and the thermal expansion polymer of U0 is much more than that of E0 and P0 under the same temperature. The friction surface of U0 can quickly form a transfer film when the steel ball began to move, and quickly achieves a stable friction stage and avoids serious wear. Meanwhile, the thermal expansion of the polymer is greater when heated by a higher temperature, which can provide more lubricant and avoids the exhaustion of the transfer film. It is said that a continuous transfer film is generated on the steel surface at 100 °C and, thus, the wear rate is low at high temperature.

### 3.4. Effects of MoS_2_ on the Friction Coefficient of E1 and U1

The above study showed that U0 and E0 had low friction coefficients and high wear resistance. Thus, UP and EP were chosen as the base polymers to fabricate MoS_2_–containing laminated composites, and the tribological performances under temperatures from 25 °C to 150 °C were discussed.

**Figure 7:**
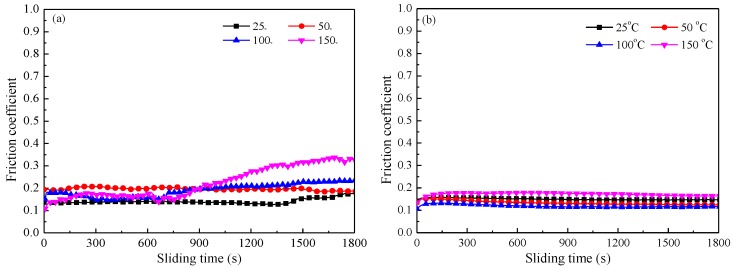
Friction coefficient of (**a**) E1 and (**b**) U1 at 25–150 °C under a load of 6 N and a sliding speed of 4 Hz.

Figure 7a plots the friction coefficient of E1 sliding against the steel ball at a load of 6 N and a sliding speed of 4 Hz. At 25 °C, the friction coefficient of E1 is 0.13 at the initial stage and then remains relatively the same for 1450 s, followed by slowly increasing and reaching 0.18 at the end of the test. When the temperature is 50 °C, the friction coefficient moderately fluctuates in the range of 0.18–0.20, while at 100 °C, the friction coefficient is 0.17 at the start-up stage, and then decreases to the minimum value of 0.15 after running for 300 s and maintaining for 350 s. Afterwards, it gradually increases to 0.23 at the end of the test. The grooved textures provide reservoirs for the lubricant under high temperature, so the friction coefficient is low and stable [9]. For temperatures as high as 150 °C, the friction coefficient increases from 0.10 to 0.17 after running for 300 s, and then remains relatively stable. After sliding for 700 s, the friction coefficient significantly increases to 0.32. This indicates that the friction coefficients of E1 increase as the temperature rises.

Figure 7b compares the friction coefficient of U1 under a load of 6 N and a sliding speed of 4 Hz. The initial friction coefficient is 0.12 at 25 °C, and it smoothly declines with prolonged sliding, finally it reaching a steady state (with a value of 0.15). At 50 °C, the friction coefficient is 0.13 at the starting stage, then it slowly decreases to 0.12 and remains stable. When the temperature is 100 °C, the friction coefficient tendency is similar to that under 50 °C, and the average friction coefficient is lowest (0.11). However, the average friction coefficient increases to 0.16 as the temperature increases to 150 °C, and the friction coefficient slowly declines with prolonged sliding.

Based on a comprehensive analysis of Figure 6 and Figure 7, it is illustrated that (i) when MoS_2_ filled into the laminated composite surface, the friction coefficients were much lower than those of the composite without adding MoS_2_; (ii) the friction coefficients of E1 are relatively stable under 25 °C–50 °C, while they have high values and wild fluctuations as the temperature increases to 100 °C and 150 °C; and (iii) the friction coefficients of U1 are very low and relatively stable under the test temperatures.

Solid lubricant stored in grooves is expanded by the external heat and the frictional heat. The solid lubricant swells to the surface and forms a transfer film on the contact area under the drag effect of the sliding ball, which results in a relatively stable and low friction coefficient [18]. When the temperature rises to 150 °C, the friction coefficients of E1 and U1 are higher than that at lower temperatures. However, the heat on the sliding surface is difficult to effectively spread out with the increase of frictional heat, the transfer film on the contact area becomes soft, and the plastic deformation of the polymer is significant, resulting in an increase of friction. In addition, thermal stress and contact stress accelerate failure of the transfer film, which also leads to the increase of the friction coefficient.

### 3.5. Wear Surface Analysis and Self-Lubricating Mechanism

The self-lubricating mechanism of the laminated composite surfaces was identified by SEM morphology and element distribution of the wear scars after the friction test.

**Figure 8:**
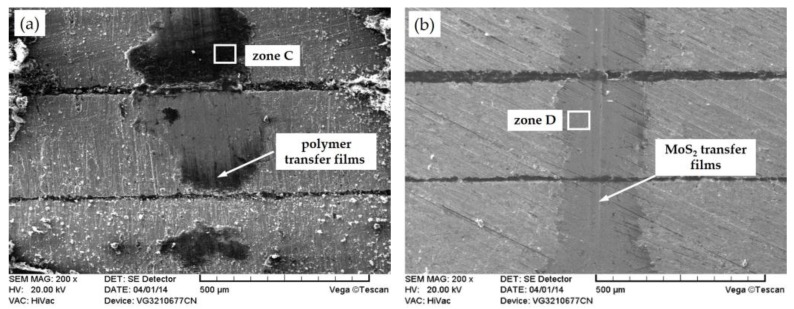
SEM morphology of (**a**) E0 and (**b**) E1 at 25 °C under a load of 6 N and a sliding speed of 12 Hz.

**Figure 9:**
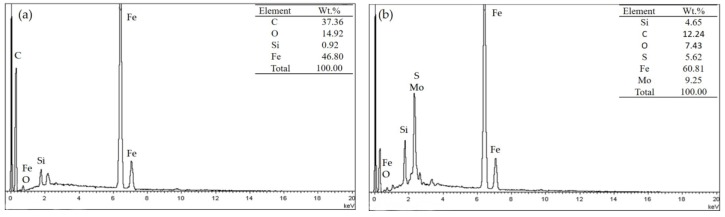
Energy spectrum analysis of the wear surface: (**a**) E0 and (**b**) E1.

Figure 8 shows the SEM morphology of wear scars on the friction surfaces of the E0 and E1 sliding against the steel ball under a load of 6 N and sliding speed of 12 Hz. As shown in Figure 8a, significant plowing grooves and wear debris on the worn surface of E0 are observed. Meanwhile, the EDS spectrum of zone C (as shown in Figure 9a) indicates that discontinuous polymer transfer films are also observed on the surfaces of the steel zones. The reason is that the lubrication of transfer film generated by polymer without solid lubricant is poor, thus, the friction coefficient and wear rate are larger and the transfer film fails easily, and the surface is very rough. With the polymer containing MoS_2_, a continuous and uniform transfer film is discovered on the wear scar (as shown in Figure 8b). In addition, the surface is smooth and no wear debris or furrows are noticed on the worn surface, because MoS_2_ can generate high-performance lubrication films on the sliding surface during dry sliding conditions [19].

The distribution of elements on the wear scars reflects the property of transfer film formed on the friction surface. Figure 9 shows the EDS spectrograms of wear scars for specimen of E0 (zone C) and E1 (zone D), respectively, after the dry sliding test. For E0, elements of C and O are found on the wear surface, indicating the pure epoxy generated transfer film on the steel surface. In comparison, elements of Mo and S are detected on the E1 surface, indicating that MoS_2_ particles moved onto the friction surface along with the thermal expansion of the polymer, which is conducive to the formation of a self-lubricating transfer film and leads to excellent tribological properties of the laminated composite containing MoS_2_. Therefore, the analysis of the elements of the wear scars shows that the lubricant filled in the grooves are transferred to the surface due to the thermal expansion, and the transfer film generated on the steel surface can separate the steel asperities from directly making contact.

**Figure 10:**
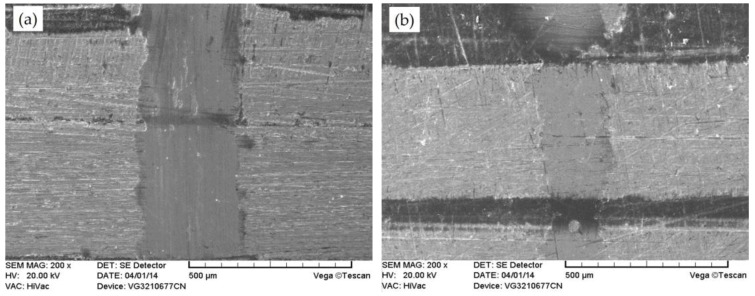

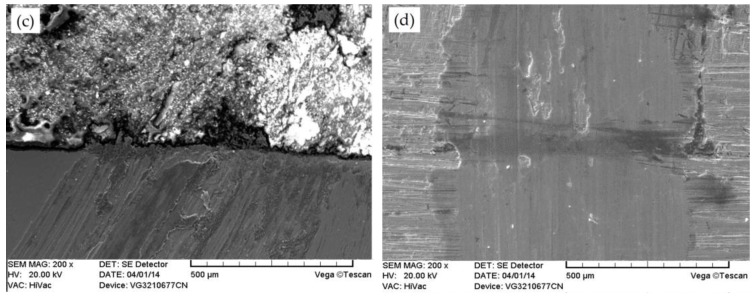
SEM images of the friction surface: (**a**) E1 at 50 °C; (**b**) U1 at 50 °C; (**c**) E1 at 150 °C; and (**d**) U1 at 150 °C under a load of 6 N and a sliding speed of 12 Hz.

Figure 10 shows the SEM images of E1 and U1 at different temperatures. After the dry sliding test at 50 °C (Figure 10a, b), the continuous transfer film is clearly seen on the wear scar of E1 and U1 surfaces. Meanwhile, the width of the wear scar on E1 is wider than that of U1, and a slight delamination is obviously found on the transfer film of E1 (as shown in Figure 10a).

When the test temperature is 150 °C (Figure 10c, d), the transfer films which formed on the worn surface of E1 are destroyed, a mass of furrows and wear debris are also exhibited on the worn surface. However, the worn surface of U1 also retained a relatively continuous transfer film and was smoother, with little delamination compared with that of E1. Meanwhile, the transfer film is wider than that at 50 °C; this it is because the polymer is softer and has a better film-forming property at higher temperature.

**Figure 11:**
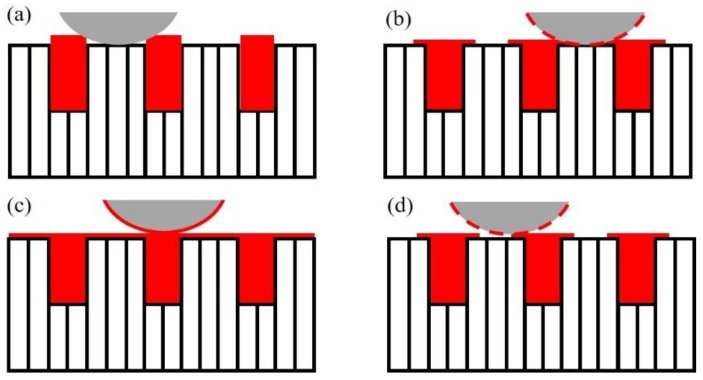
Self-lubricating mechanism: (**a**) thermal expansion of the solid lubricant; (**b**) formation of the local transfer film; (**c**) formation of the continuous transfer film; and (**d**) failure of the transfer film.

The self-lubricating mechanism is a dynamic process that: the polymer was expanded from the grooves under frictional heat and a thermal-field and rolled on the friction surface, and the formation and failure of the transfer film varied with the sliding time. The schematic diagram of the self-lubricating mechanism is shown in Figure 11.

In the initial stage, the friction coefficient is high as the steel asperities are in direct contact with each other, which induces a large amount frictional heat generated on the contact surface. The thermal expansion coefficient of the polymer is much higher than that of the steel sheet [14], and the polymer expands to the frictional surface by thermal stress (as shown in Figure 11a). In the following, polymer adheres on the surface and forms a discontinuous transfer film under the crush of a steel ball. With prolonged friction testing or under a higher temperature field, an abundant solid lubricant is thermally expanded and forms a continuous transfer film (as shown in Figure 11b) and, thus, the friction coefficient can be significantly reduced. However, micro-cracks may be generated on the transfer film under the cyclic loading of the steel ball, and this induces failure of the film and increases the friction coefficient and deteriorates the surface wear, resulting in serious wear of the transfer film between the pairs, and the self-lubricating of the laminated composite surface may fail (as shown in Figure 11d).

## 4. Conclusions

Three kinds of resins were used to fabricate laminated composite surfaces by the lamination-bonding process based on the bionics principle. The tribological properties of the laminated composites were conducted under different temperatures, and the effects of MoS_2_ on the mentioned performances were discussed. The following conclusions are drawn:

- For the laminated composite surface without adding solid lubricant, the friction coefficient and wear rate of E0 and P0 are much higher than that of U0. Meanwhile, the friction coefficients of the laminated composite are obviously low at a high sliding speed of 12 Hz and high temperature of 100 °C, respectively.
- The friction coefficient of the laminated composite containing MoS_2_ is much lower than that without adding MoS_2_ as the temperature increases. However, at a temperature of 150 °C, the friction coefficient of E1 is increased to 0.35, while that of U1 is stable at 0.17.
- MoS_2_ polymer filled in the grooves thermally expand under a high temperature field and, thus, the MoS_2_ particles generate a transfer film on the sliding surface which endows the good tribological performance of the laminated composite under high temperature. However, the transfer film is consumed at an accelerated rate as temperature increases, which may induce the fatigue of the self-lubricating laminated composite.

## Figures and Tables

**Table 1:** Formulation of self-lubricating polymer composites.

Sample	Resin (g)	Curing Agent (g)	MoS_2_ (g)
E0 (Epoxy resin)	10.0	1	0
P0 (Phenolic Resin)	10.0	1	0
U0 (Unsaturated polyester)	10.0	0.5	0
E1 (Epoxy resin)	10.0	1	4
U1 (Unsaturated polyester)	10.0	0.5	4

**Table 2:** Tribological test conditions.

Item	Value
Applied load (N)	6
Sliding frequency (Hz)	4 and12
Stroke (mm)	6
Sliding distance (m)	43.2 and 129.6
Diameter of the steel ball (mm)	9.5
Lubrication type	Dry
Test time (min)	30
Temperature (°C)	25–150
Relative humid (%)	40–45
